# Natural Mosquito-Pathogen Hybrid IgG4 Antibodies in Vector-Borne Diseases: A Hypothesis

**DOI:** 10.3389/fimmu.2016.00380

**Published:** 2016-09-29

**Authors:** Berlin Londono-Renteria, Jenny C. Cardenas, Andrea Troupin, Tonya M. Colpitts

**Affiliations:** ^1^Department of Pathology, Microbiology and Immunology, University of South Carolina School of Medicine, Columbia, SC, USA; ^2^Clinical Laboratory, Hospital Los Patios, Los Patios, Colombia

**Keywords:** IgG4, bi-specific, arthropod saliva, vector-borne diseases, malaria, dengue virus, mosquito

## Abstract

Chronic exposure to antigens may favor the production of IgG4 antibodies over other antibody types. Recent studies have shown that up to a 30% of normal human IgG4 is bi-specific and is able to recognize two antigens of different nature. A requirement for this specificity is the presence of both eliciting antigens in the same time and at the same place where the immune response is induced. During transmission of most vector-borne diseases, the pathogen is delivered to the vertebrate host along with the arthropod saliva during blood feeding and previous studies have shown the existence of IgG4 antibodies against mosquito salivary allergens. However, there is very little ongoing research or information available regarding IgG4 bi-specificity with regard to infectious disease, particularly during immune responses to vector-borne diseases, such as malaria, filariasis, or dengue virus infection. Here, we provide background information and present our hypothesis that IgG4 may not only be a useful tool to measure exposure to infected mosquito bites, but that these bi-specific antibodies may also play an important role in modulation of the immune response against malaria and other vector-borne diseases in endemic settings.

## Pathogens and Mosquito Saliva – A Brief Introduction

Infectious diseases are one of the leading causes of mortality worldwide (1, 2). From this large group, the vector-borne diseases are among the leading causes of mortality and disability in developing countries (3, 4). The majority of arthropod-borne diseases lack a specific vaccine; thus, prevention relies predominantly on vector control interventions (5, 6). In the last decade, considerable effort has been put toward the dissection of arthropod factors that modulate the transmission of pathogens (7–9). Several studies have shown that during transmission, vector saliva plays an important role in the establishment of a successful infection and favors the pathogen survival by modulating the local immune responses (7, 10–14). Most salivary proteins (SPs) are highly immunogenic, able to elicit antibody production and memory responses (15, 16). During probing and feeding, mosquitoes deposit SP in the vertebrate host to facilitate the blood meal intake (17–19). The immune response induced by arthropod saliva is mainly Th2, which favors the production of antibodies (20, 21). IgG4 antibodies are known to reflect repeated exposure to environmental antigens and allergens (22, 23). Additionally, recent research by our laboratory has found that exposure to mosquito saliva can be correlated with disease clinical presentation (15, 24, 25). Chronic exposure to mosquito bites induces higher IgG4 antibodies against SPs than other IgG subclasses (22, 26, 27), suggesting that this specific antibody subclass may be a marker of intense exposure to arthropod vectors (28, 29). Here, we present a brief review on the antibody response against SPs and the hypothetical implication of IgG4 antibodies in disease progression of several vector-borne diseases.

## IgG4: A Special Molecule

As a general characteristic, IgG4 is an effective immuno-regulator (23). This is due to the fact that, although IgG4 may act as a blocking antibody, it is not efficient in forming large immune complexes (30). IgG4 antibodies are able to interact with the receptors FcγRI, FcγRIIA, FcγRIIB, FcγRIIC, and FcγRIIIA. Interestingly, the inhibitory receptor FcγRIIB has lower affinity for IgG1, IgG2, and IgG3 than the other Fc receptors (FcRs), but affinity is not lower for IgG4 (31). This interaction may be in part responsible for the anti-inflammatory properties of IgG4. In addition, IgG4 antibodies block IgE-mediated inflammatory responses by competing for antigen recognition sites, thus inhibiting Fcε-receptor cross-linking and further signaling (32, 33). IgG4 is the only IgG subclass with equal affinity for activating FcRs and for the inhibitory receptor FcγRIIB (34). If IgG4 co-interact with both inhibiting and activating receptors, the result would be inhibition of effector cell responses (34, 35).

Naïve B cells express a monomeric membrane-bound B cell receptor normally as IgM or IgD antibodies (36, 37). After activation of such B cells and aided by specific cytokines, rearrangement of the antibody heavy chain locus through class switch recombination results in the expression of a new isotype (i.e., IgG). In the case of IgG4, production relies on the Th2 cytokines IL-4 and IL-13 (38). The Th2-associated cytokine, IL-10, is a key factor in the activation of IgG4 producing B cells (39). Secretion of IL-10 will skew immune response to the production of IgG4 antibodies over other IgG subclasses. IL-10-producing B cells regulate pro-inflammatory immune responses and a lack of them can lead to exacerbated chronic inflammation (40, 41). Previous studies showed that IgG4 may be selectively produced in human B regulatory 1 (BR1) cells and that allergen-specific B cells present increased expression of both IL-10 and IgG4 (42). Distinctive from other IgG subtypes, IgG4 is the only antibody class that is able to recognize two antigens of different nature; therefore, it is often termed a bi-specific antibody (23). A requirement for this *bi-specificity* is that both eliciting antigens must be present at the same time and at the same place when the immune system is stimulated (43). This is the case of naturally acquired infections transmitted by arthropods where pathogens, such as parasites or viruses are delivered into human skin imbibed in saliva with SP (44).

**Figure 1:**
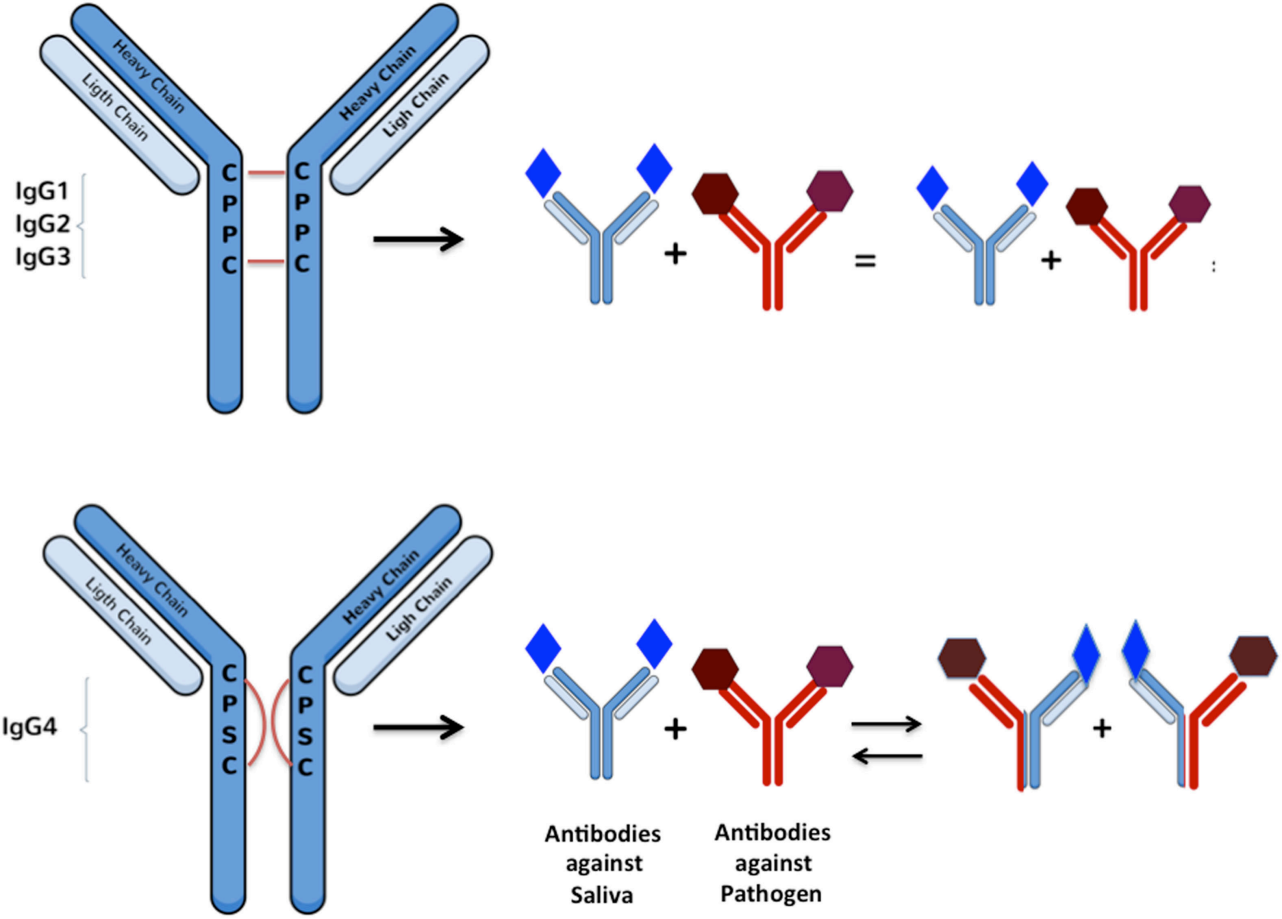
**Schematic representation of Fab-arm exchange between IgG molecules: changes in heavy chain sequences makes the inter-chain interactions weaker and favoring intra-chain interactions in a way that allows arm disassembly and further rearrangement between molecules recognizing antigens of different nature**. This reaction can be simulated *in vitro* under reducing conditions with glutathione.

IgG4 molecules are synthesized the same way as all the other IgG subclasses (23). However, extensive research has demonstrated important sequence differences among all IgG subclasses that confect various levels of stability between chains in the hinge region (45, 46). These studies shown that one single substitution of a proline for an serine in the light chain allows IgG4 molecules to undergo a process called the “half-molecule exchange” where one antibody molecule directed against a specific antigen has the capacity of “exchanging” a half-molecule (a heavy chain and an attached light chain) with another IgG4 molecule synthetized against another non-related antigen (45–48) (Figure 1). Normally, a single B cell will produce IgG molecules with identical copies of a heavy chain determining the immunoglobulin isotype (e.g., G) and two identical copies of light chains (κ or λ) (49, 50). During the half-molecule exchange process, hybrid IgG4 molecules could contain two heterogeneous light chains (i.e., κ/λ instead of κ/κ or λ/λ). A recent publication described that hybrid bi-specific IgG4 κ/λ antibodies represent approximately up to 30% of the total IgG4 antibodies in human sera (51). Unfortunately, the exact mechanism involved with the *in vivo* generation of specific IgG4 molecules is largely unknown.

**Figure 2:**
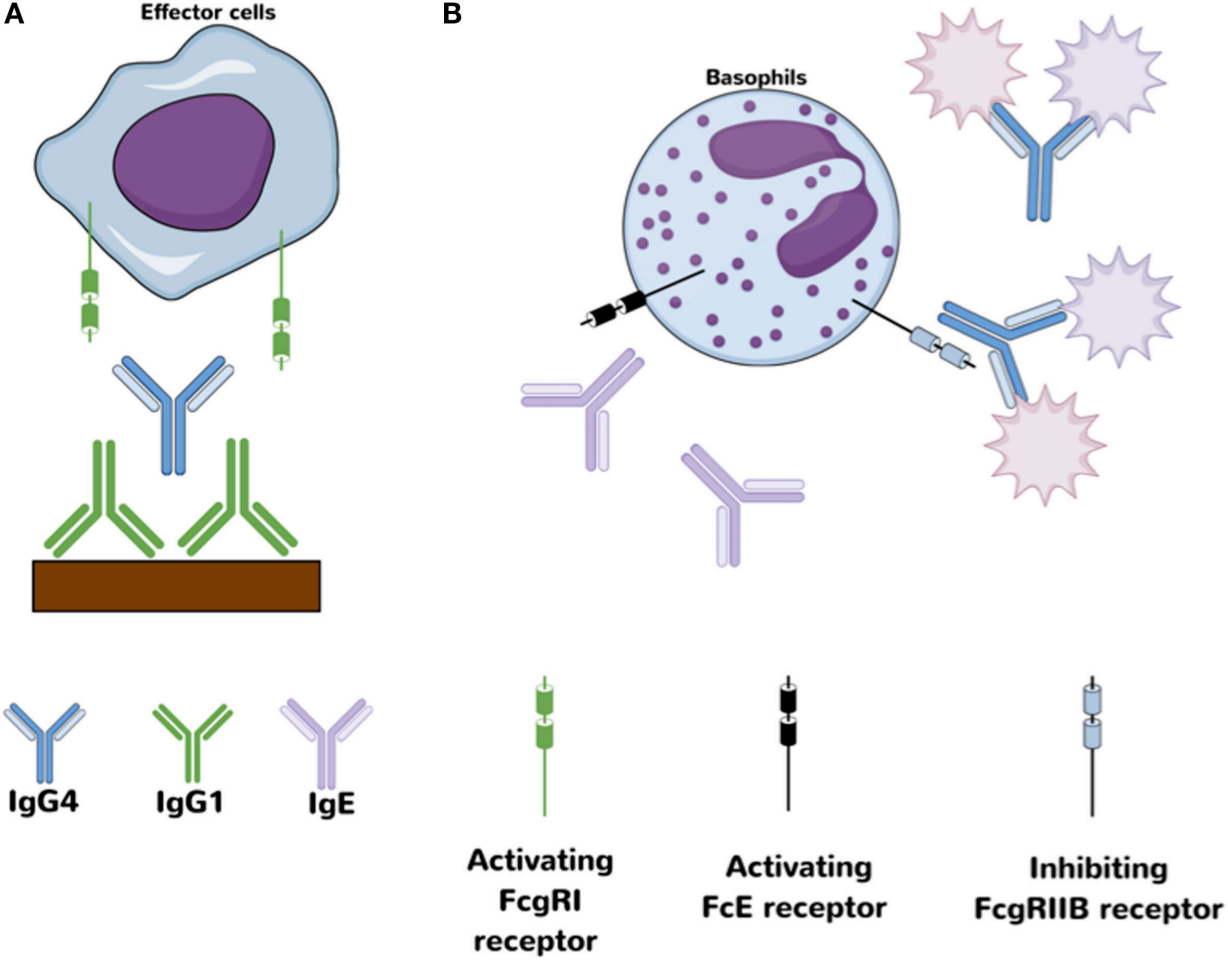
**Proposed anti-inflammatory mechanism for IgG4 antibodies**. **(A)** Fc–Fc interaction between IgG4 and IgG1 may block access to activating Fcγ receptors on effector cells. **(B)** Antigen-binding competition against IgE avoids/decreases cross-link with activating Fcε receptors.

Another special characteristic of IgG4 antibodies is their ability to interact with other IgG subclasses, particularly IgG1, in such a way as to alter/block their functionality and FcR binding (48). Specifically, previous studies have found that the Fc portion of IgG4 is able to interact with other IgG antibodies in an Fc–Fc fashion due to conformational changes that allows interaction between CH3 motifs from both molecules (45, 48). This interaction is specially favored when the target, IgG1 or another IgG4 molecule, is coupled to a solid phase (Figure 2). Fc–Fc interactions between two IgG4 may potentially be an intermediated step in the formation of bi-specific IgG4 molecules, allowing the contact and further recombination of two IgG4 molecules. A more detailed description of the Fc–IgG4 interactions with other Fc–IgG and their potential implication *in vivo* is explained in detail by Rispens et al. (48) and Davies et al. (45).

In the case of vector-borne human diseases, anti-SP IgG4 may undergo this half-molecule exchange with IgG4 molecules against a pathogen. Since IgG4 antibodies are often present in individuals who are chronically exposed to mosquito saliva allergens, we hypothesize that some of these antibodies may form hybrid bi-specific molecules with pathogen-specific IgG4 during natural transmission of vector-borne diseases. This hypothesis is also based on the fact that mosquito saliva alone is able to increase the level of IL-10 in exposed tissues creating a favorable environment for the production of IgG4 antibodies against the antigens present. If those antibodies are against two different antigens, the half-arm exchange may be induced.

The majority of research on the characteristics of bi-specific antibodies has been focused on artificial antibodies for cancer treatment or the role of natural bi-specific antibodies in autoimmune diseases, such as rheumatoid arthritis (RA) (52–54). The mechanisms involving IgG4 in autoimmune diseases is still not understood, but findings suggest that the presence of natural bi-specific antibodies in serum from RA patients may serve as indicators for disease remission (53). Additionally, artificial bi-specific antibodies are among the most promising treatment choices for cancer and inflammatory-mediated diseases over monoclonal antibodies because they can simultaneously target two different antigens and improve therapeutic outcomes (55, 56). Two options of artificial bi-specific antibodies are already available in the market and several others are in clinical trials (55). However, there is a stark lack of research with regard to infectious diseases transmitted by arthropods and the participation, if any, of bi-specific IgG4 in the immune response against them. We believe that possible missing clues in vaccine or/and treatment development for malaria and other vector-borne diseases may be hidden within the properties of this double-handed molecule known as IgG4.

**Figure 3:**
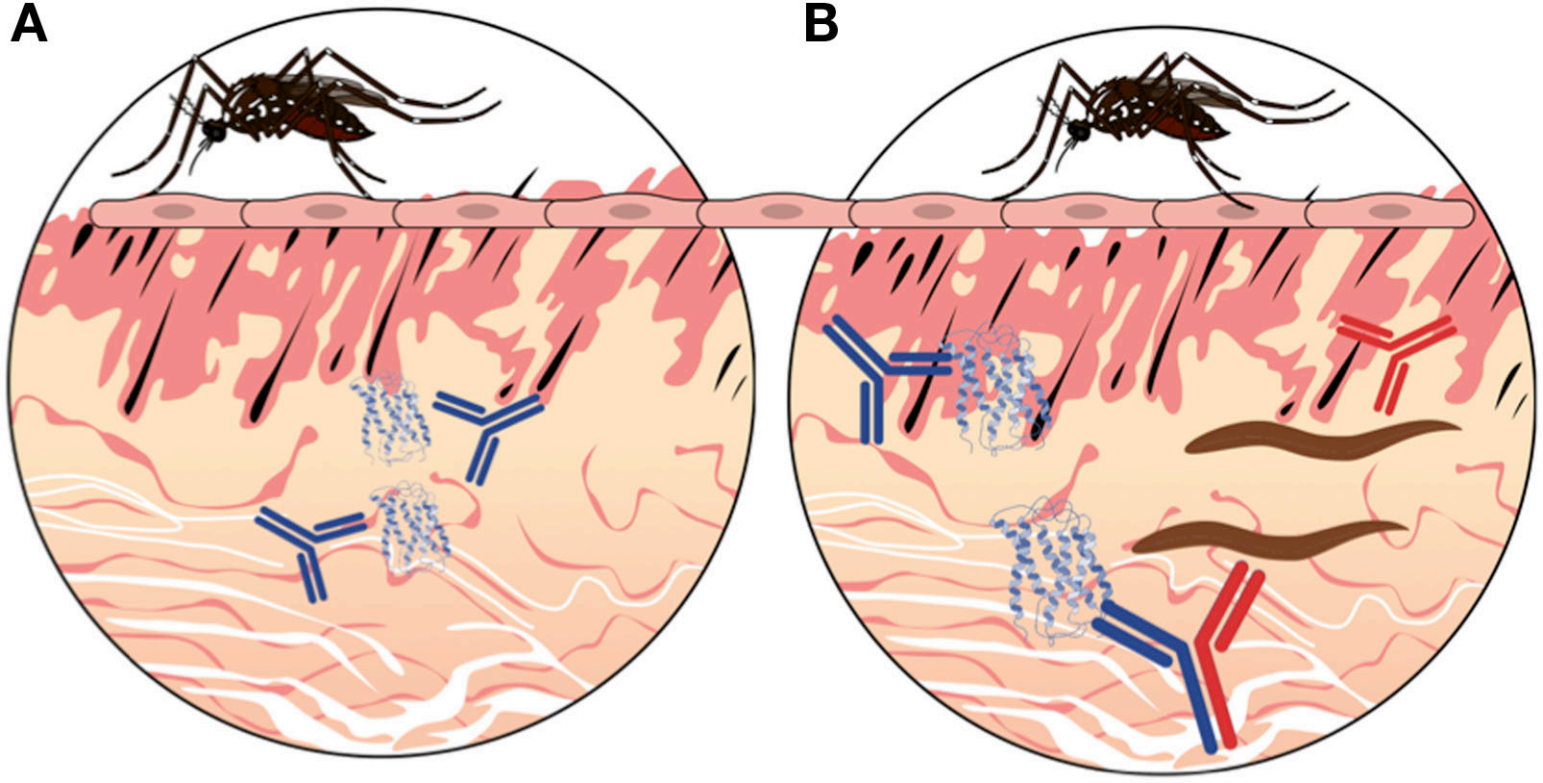
**A hypothetical representation of the hybrid IgG4 formation in vector-borne diseases**. **(A)** In the absence of infection in the mosquito, probing will inject saliva proteins into the vertebrate host during blood feeding. Thus, antibodies will be made against mosquito proteins only (BLUE). **(B)** In the presence of infection in the mosquito, the pathogen will be delivered in the skin along with the salivary proteins. Antibodies will be made that recognize mosquito proteins (BLUE) as well as pathogen proteins (RED). The chronic exposure to different antigens will stimulate the production of IgG4 molecules that will undergo fab-arm exchange when two different antigens are present, resulting in bi-specific antibody molecules (BLUE AND RED).

Bi-specific IgG4 molecules are stimulated in an environment of diverse antigens. For example, previous studies have shown that a single IgG4 molecule is able to bind grass/pollen antigens and house dust mite antigens at the same time (43). In the case of allergens, such as mosquito saliva, which is composed of a diverse cocktail of molecules, the IgG4 bi-specificity could be elicited against the various salivary components producing “SP–SP” bi-specific IgG4 (Figure 3A). However, in the presence of pathogen/saliva mixture (delivered via an infective insect bite), a proportion of “pathogen-SP” IgG4 would likely be produced (Figure 3B). Indeed, chronic exposure to mosquito salivary antigens has been proposed to play a role in immunity against chronic versions of several vector-borne diseases, such as filariasis, leishmaniasis, and malaria (57–59), by stimulating the production of pathogen-SP IgG4 bi-specific antibodies.

The human immune response against vector-borne pathogens often involves the production of IgG1 antibodies with high pro-inflammatory activities that are able to clear most pathogens. If conformational changes are induced at the Fc portion of an antigen-bound IgG1 molecule, a significant IgG4-mediated response against SPs and/or pathogen antigens may block an IgG1-mediated response, which would have a profound effect on the fate of infectious disease progression and immune response Thus, IgG4 and bi-specific IgG4 antibodies may represent attractive examples of how the immune response to environmental antigens could potentially modulate the fate of infectious diseases in humans (35).

## IgG4 and Parasitic Infections

### Filariasis

Human filariasis (lymphatic filariasis, Onchocerciasis, and Loiasis) is group of parasitic infections often transmitted by arthropods to humans and animals (60–62). With the exception of *Dracunculus medinensis*, all filarial worms use insects as intermediate hosts (63). These diseases are important causes of disability and physical deformity in developing countries (62). It is calculated that at least one-fifth of the global population is at risk and there is not any commercially available vaccine (64). However, in spite of challenges in program coverage and compliance, significant progress has been made in reducing the incidence of filarial infections by mass drug administration programs (65).

As in the rest of human helminthiasis, IgG4 antibody response is the hallmark of filarial infections. Previous studies have shown that up to 95% of IgG antibodies against filarial worms belong to the IgG4 subclass (66, 67). Filarial parasites preferentially induce the production of IgG4 antibodies to evade immune response in the infected host; thus, filarial infections are often chronic (68). Adjobimey and Hoerauf (67) suggested that the immune regulatory properties of IgG4 in filarial infection could be associated with the half-molecule exchange property of this antibody (67). Mosquitoes from the genus *Aedes, Anopheles, Culex*, and *Mansonia* can all transmit filarial parasites. Whether bi-specific antibodies are elicited against salivary/pathogen antigens in filariasis or other helminthic diseases remains to be investigated, though it seems a likely scenario.

Previous studies demonstrate that the levels of IgG antibody subclasses during infection depend on filarial transmission intensity. In higher transmission areas, antigen-specific IgG4 levels against different parasite stages (microfilaria versus adults stages) are associated with parasite carriage and asymptomatic infection (68, 69), In fact, people with asymptomatic filarial infection present significantly higher IgG4 antibody levels than people with severe disease. By contrast, lower IgG4 antibody levels, along with high titers of IgE, are present in severe cases (70–72). It is proposed that IgG4 antibody levels in filarial infected patients is associated with the blocking effect of the IgE mediated, often exacerbated, pro-inflammatory responses (61).

### Malaria

Malaria is currently the most prevalent parasitic disease transmitted by mosquitoes in the world; it is caused by parasites of the genus *Plasmodium* (73). Several *Plasmodium* species can cause disease in human but it is *Plasmodium falciparum* that is the single species responsible for majority of human deaths (74, 75). In spite of major significant advances in the knowledge of malaria transmission and several vaccine trials in recent years, there is still not a single vaccine against *Plasmodium spp*. infection commercially available (76). A vaccine against malaria is desperately needed to prevent the nearly 500,000 deaths from this disease each year, most of them children under the age of 5 years (77–79). Interestingly, in endemic areas, natural “protective immunity” is developed over decades of exposure to infected mosquito bites (80). People who develop this type of immunity are partially protected against severe disease and more dangerous symptoms. This would mean that the longer someone lives in an endemic area, the more protected the individual would be against severe malaria. In fact, the association between asymptomatic malaria carriers and age has been well documented (81–85). Unfortunately, this immunity is short-lived and may wane once the person leaves the endemic, mosquito-rich area or when exposure becomes less constant (i.e., travel, emigration) (80, 86). When this happens, the body returns to a “naïve” state and the person may develop symptoms, and even severe disease, upon a new infection with *Plasmodium* (87).

In humans, immune response against malaria seems to be mediated by “cooperation” between cellular and antibody-mediated responses. The main antibodies associated with protective immunity against malaria are the IgG subclasses, IgG1 (88, 89). Since IgG4 may interfere with the activity of other antibody subclasses, we hypothesize that IgG1-mediated responses against *Plasmodium* could be influenced by an increase in IgG4 antibodies induced by the presence of saliva. These IgG4 antibodies may bind *Plasmodium*-bound IgG1 antibodies blocking Fc-receptor-mediated responses, opsonization and/or complement activation, consequently, enhancing parasite survival. It is also possible that, in the presence of saliva, bi-specific *Plasmodium–Anopheles* IgG4 antibodies are induced and play a role in developing protective/detrimental immune responses during malaria infection.

One of the main SPs in the principal malaria vector in Africa, *Anopheles gambiae*, is the salivary gland protein 6 (gSG6), a small proteins exclusively expressed in adult female salivary glands that plays a role blood feeding (90). Antibodies against a peptide derivative from this protein, the gSG6-P1, have been validated as a marker for *Anopheles* bite exposure (91, 92). The gSG6 protein is recognized by IgG4 antibodies in serum from people chronically exposed to *Anopheles* bites (27, 93). Although the presence of bi-specific IgG4 antibodies or a direct correlation between gSG6-IgG4 and *Plasmodium* IgG1 has not yet been evaluated, a recent study suggested a negative effect of mosquito bite exposure on the IgG1-mediated immune response against malaria. This study showed that children with higher levels of exposure to *Anopheles* bites presented lower IgG1 responses against *Plasmodium* antigens and parasitemia was significantly correlated with IgG1 antibody levels (94), suggesting an impact of immunity against SPs in the immune response against infection in pediatric populations. The impact of anti-salivary immunity in adults was not described in this study. It is possible that high exposure to mosquito bites in children can induce anti-SP IgG4 antibodies capable of binding *Plasmodium-*specific IgG1 and block its activity; therefore, resulting in more symptomatic disease. However, extensive further research is needed to evaluate whether there is a correlation between the anti-SP IgG/IgG4 ratio and symptomatic disease in children as well as whether bi-specific antibodies play a role in age-dependent protection/pathogenesis in malaria.

### *Leishmania* 

Leishmaniasis is a group of parasitic diseases [cutaneous, mucocutaneous and visceral leishmaniasis (VL)] caused by parasites of the genus *Leishmania spp*. Leishmaniasis is transmitted by *Phlebotomus spp*. (Old World) and *Lutzomyia spp*. (New World) sandflies (95, 96). Cutaneous leishmaniasis (CL) is often presented as chronic lesions developed from months to years. Previous studies have shown an association between disseminated CL forms of leishmaniasis and IgG4 antibody levels (97). In fact, proper healing is often associated with a Th1-mediated response while disseminated disease is associated with Th2 responses. CL and mucocutaneous leishmania (ML) infections are characterized by successively higher specific antibody titers that reflect the length of infection and parasite loads (98). High antibody titers can also be detected in VL due to a polyclonal activation of B cells (99). VL is a serious parasitic disease caused by the protozoan *Leishmania donovani* (Asia) and *L. infantum/L. chagasi* with more than 200 million people at risk globally (95, 100). The Post-kala-azar dermal leishmaniasis (PKDL) is a complication presenting in patients infected with *L. donovani* that have recovered from VL (101). It has been found that IgG4 is significantly elevated in PKDL compared to VL, especially in pediatric populations (102). Higher IgG4 antibody levels have also been observed in people with active VL in comparison to treated patients or those with subclinical disease (103). Additionally, a decrease in the IgG4 antibody titer has been observed in children with VL after treatment, suggesting that IgG4 may be a suitable immunological marker for the assessment of drug treatment (104).

It has been well documented that saliva from sandflies enhances Leishmania infection during the first stages of the infection. This is thought to be due to the immunosuppressive effect of several saliva components on macrophages and T cells (105–108). Interestingly, immunity against sand fly salivary molecules may confer protection against disease (108, 109). In mouse models, the protection observed in pre-exposed animals to sandfly saliva involved a strong induction of Th1 response and delayed-type hypersensitivity (DTH) response reaction against saliva that creates a detrimental environment for the parasite (110). These studies showed that the immune response against the phlebotomine sandfly’s SPs has modulatory effects on disease pathogenesis and some of these proteins are considered candidates for vaccine or drug synthesis (111, 112). It is hypothesized that the study of mosquito salivary factors with similar activity could lead to novel candidates for other arthropod transmitted diseases (11, 113). In addition, previous studies have shown that people exposed to sandfly bites present specific antibodies against the saliva of the main vectors in the area where they reside and little or no cross-reactivity to other sandfly species. People with active leishmaniasis present significantly higher IgG antibody levels against SPs of the main vector when compared with healthy individuals living in the same area (114).

One study demonstrated that IgG4 antibodies are the main antibody subclass against SPs of the *Ph. papatasi* sandfly (115). It has been shown that previous exposure to the American sandfly *Lu. intermedia* can induce a significant increase in IL-10 expression resulting in exacerbation of infection after challenge with *Leishmania braziliensis* (116, 117), although positive correlation between antibody response to saliva and cellular response to Leishmania has not been reported. Importantly, individuals seropositive to saliva are twice more likely to develop CL (118). Infection by *L. braziliensis* is responsible for the majority of ML characterized by highly destructive mucosal lesion. ML often follows skin lesions even decades before the mucosal involvement (119). In that time, it is very likely that people continue being exposed to vector bites, which may induce the production of bi-specific IgG4 antibodies against mosquito proteins and pathogen proteins.

## Arboviruses: A Focus on Dengue and Antibody-Mediated Responses

Dengue is a disease caused by dengue virus (DENV), which is transmitted by *Aedes* mosquitoes in tropical and subtropical regions (4). While the majority of DENV infections result in little or no disease, a small proportion of cases progresses to severe forms: dengue with warning signs and severe dengue also known as dengue hemorrhagic fever (DHF) and dengue shock syndrome (DSS) (120). It is thought that cross-reactive but sub-neutralizing IgG antibodies developed during the first DENV infection may enhance a second infection with a different serotype in a phenomenon termed antibody-dependent enhancement (ADE) (121–123). During ADE, it is thought that these sub-neutralizing antibodies may increase viral load by enabling entry into immune cells via the Fc gamma receptor (FcγR) (124). ADE is a significant risk factor for severe dengue fever (125, 126). Since IgG4 molecules are able to interact with different FcγR (31), it is imperative to study whether bi-specific IgG4 antibodies play a role in ADE.

Currently, considerable research in DENV pathogenesis is focused on pathogen-induced disease severity (127–130). Although this is of relevance, there is also a need for studies on the role of arthropod vector factors involved in pathogen infectivity and disease development. Interestingly, previous studies have shown that pathogen infection of mosquito salivary gland induces changes in the composition of saliva (131, 132). Recent studies have also shown a significant correlation between the antibody levels against these upregulated mosquito SPs and exposure to disease; thus, these proteins have been proposed as potential markers of exposure to infective bites (131). We speculate that a higher proportion of bi-specific IgG4 antibodies could be elicited against these upregulated proteins along with the pathogen, since these proteins are in higher concentration in the presence of the pathogen.

## Salivary Protein IgG4-Mediated Pathology

Previously, studies have demonstrated that bi-specific IgG4 antibodies are naturally produced in autoimmune diseases and other IgG4-related diseases (53, 54, 133). One autoimmune disease related to IgG4-mediated responses is the Fogo Selvagem (FS). In patients with FS, pathogenic autoantibodies of the IgG4 class react against desmoglein1 (Dsg1), a transmembrane glycoprotein component of vertebrate epithelial cells (134). It has been demonstrated that FS incidence overlaps significantly with exposure to *Lu. longipalpis* sandflies in Brazil (135). Interestingly, the antibody response against one specific sandfly protein, LJM11, is associated with an IgG4-mediated disease characterized by an IgG4 cross-reactivity between two antigens of different nature. It was also shown that antibodies recognizing Dsg1 during FS disease also recognize the SP LJM11, and that antibodies recognizing LJM11 can cross-react with anti-Dsg1 monoclonal autoantibodies derived from FS patients. The authors suggest that insect bites may induce a cross-reactive IgG4 antibody response, which then leads to FS disease. Their findings demonstrate a relationship between a non-infectious antigen (environmental) and the development of an autoimmune disease (136). Although the presence of bi-specific IgG4 was not evaluated, these findings suggest the potential involvement of vector saliva IgG4-mediated-pathology.

## Non-Vector Borne Chronic Viral Diseases and IgG4

In general, viral infections induce the production of neutralizing IgG1 and IgG3 antibodies over IgG4 (137, 138). However, it is possible that viruses responsible for chronic infections may also induce significant levels of IgG4 antibodies. This could be the case with infections by hepatitis B or C viruses, cytomegalovirus, Epstein–Bar virus (EBV), and human immunodeficiency virus (HIV), among others. Unfortunately, the role of IgG4 in the previously mentioned diseases has been rather neglected and conflicting results are found in the literature. For instance, in the case of HIV, one study found insignificant differences in IgG4 levels between HIV-1 positive patients and healthy controls (139), while other studies have shown that IgG4 antibody levels are significantly lower in HIV-1 patients (140–142). In addition, progression to AIDS may have a negative impact on IgG4 antibody concentration (143) since rapidly progressing patients present higher antibody levels when compared to slowly progressing individuals (141). Another study described a Th2 response characterized by high IgG4 antibody levels in children with advanced HIV-1 infections, which is thought to contribute to disease progression (144). By contrast, infection with EBV has been previously associated with IgG4-related lymphadenopathy with an increase in the number of EBV-infected cells, suggesting a direct association between EBV and IgG4-related disease (145–147) The specific mechanism involving these relationships are still under investigation. Consequently, extensive further investigation is needed to characterize the role of IgG4 antibodies in viral chronic diseases as well as to determine the existence of hybrid IgG4 molecules recognizing different viral antigens or tissue-viral antigens.

## The Hypothesis and Preliminary Data

**Figure 4:**
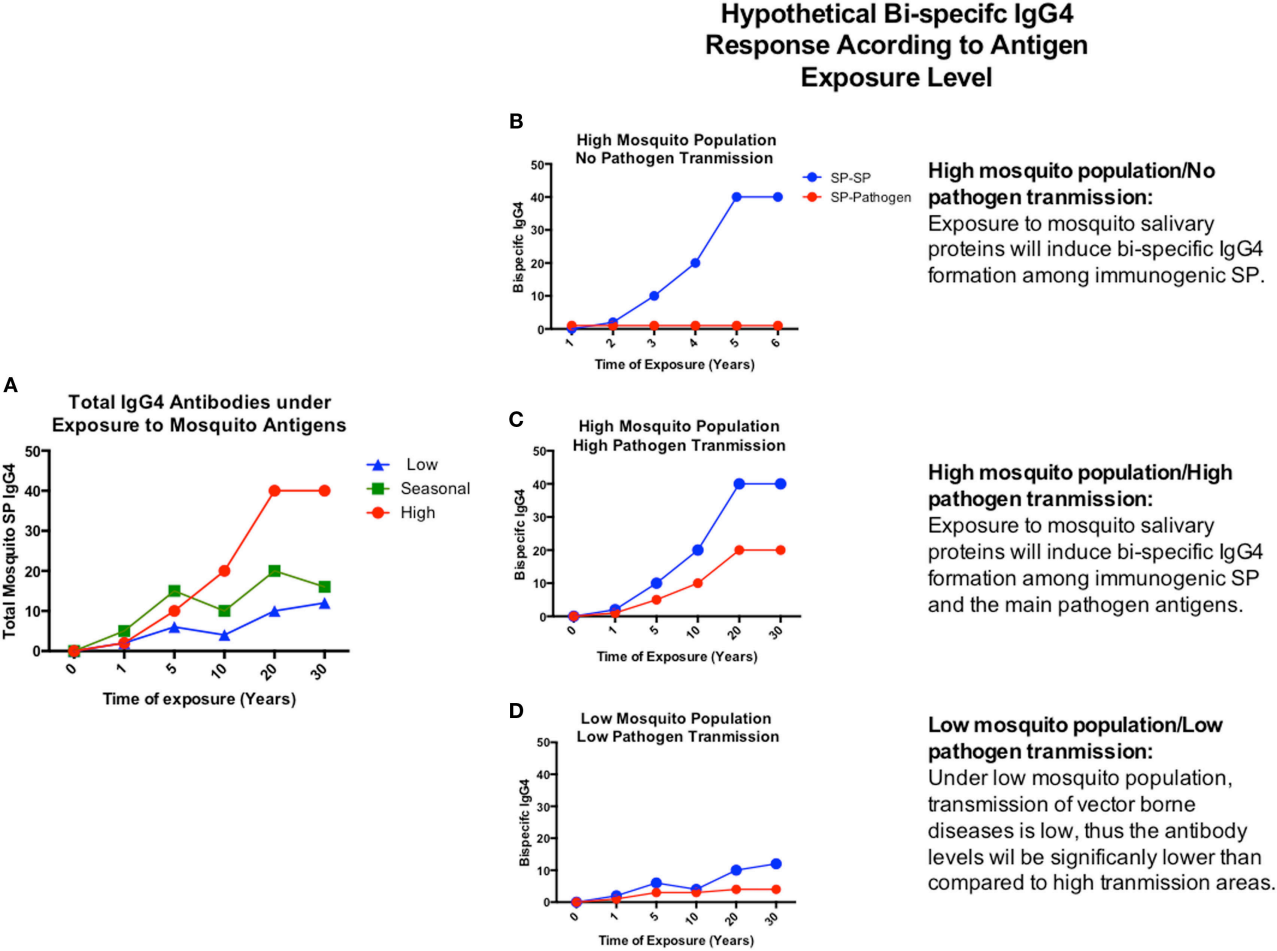
**Hypothetical chronic progression of total and bi-specific IgG4 antibodies according to disease transmission settings**. **(A)** Total IgG4 antibodies develop under chronic exposure to antigens. **(B–D)** Graphic representation of the bi-specific IgG4 antibody levels in the presence of exposure to high mosquito population/no pathogen transmission **(B)**, high mosquito population/high pathogen transmission **(C)**, and low mosquito population/low pathogen transmission **(D)**. Antibody levels will depend on the time and magnitude of exposure to antigens.

**Figure 5:**
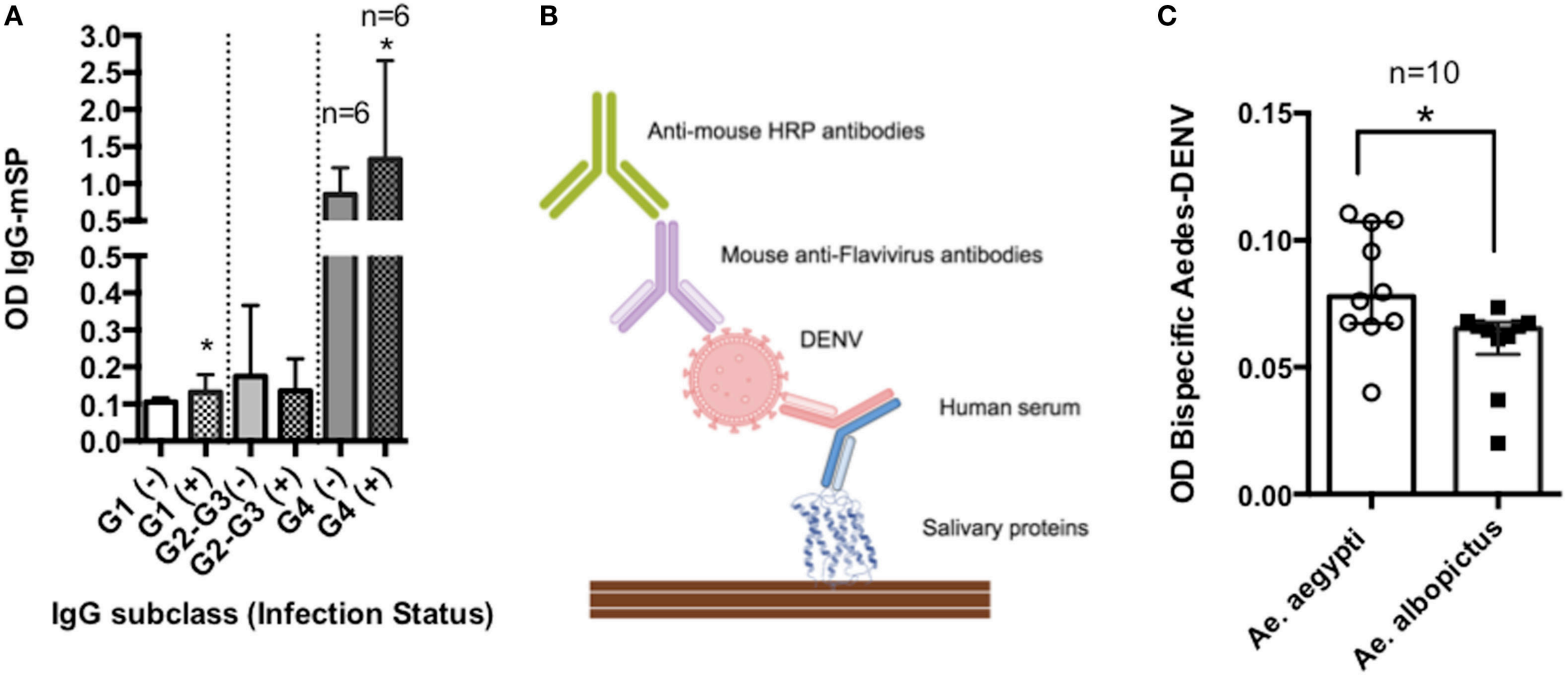
**Bi-specific IgG4 molecules in DENV infection**. Serum samples were obtained from febrile patients seeking dengue fever diagnosis. The presence of antibodies binding mosquito salivary proteins (SPs) and DENV antigen was evaluated in an ELISA-based test: plates were coated with *Ae. aegypti* SP and incubated with human serum. After washing, DENV particles were added to the plates followed by incubation with an anti-Flavivirus HRP labeled antibody. Optical densities (OD) were read at 450 nm. **(A)** IgG antibody subclass distribution in febrile individuals with (+) and without (−) active viremia. **(B)** Graphical representation of ELISA methods. **(C)** Bi-specific antibody levels against SP of the main *Aedes* species in the area. The inclusion of human subjects to evaluate the immune response against mosquito saliva was reviewed and approved by the IRB committees at the University of Pamplona (Colombia).

In a given area, people may be exposed to hundreds of mosquito bites from different mosquito species (148). Previous studies have shown that levels of anti-SP IgG4 antibodies may increase with age and they are dependent of the transmission intensity (28, 29, 149) (Figure 4A). Chances for bi-specific IgG4 antibodies to be randomly generated, increases with time and exposure intensity (Figures 4B–D). Furthermore, arthropod saliva contains dozens of proteins, each one eliciting different responses in a given host (150). Thus, we decided to test the hypothesis that virally infected mosquito saliva is able to induce the production of bi-specific IgG4 antibodies using serum from people exposed to DENV and *Aedes* mosquito bites. For this pilot test, we selected 30 serum samples from DENV positive (*n* = 24) and control (*n* = 6) human subjects living in an area where *Ae. aegypti* is the main vector of DENV, and *Ae. albopictus* was recently introduced. Samples were tested for the presence of IgG4 antibodies in an ELISA-based assay (Figure 5A). Our results demonstrate that the main antibody subclass against *Ae. aegypti* SP is the IgG4, consistent with previous studies (22, 71). We next examined whether these IgG4 antibodies are bi-specific by testing their ability to bind both DENV antigens and *Aedes* SP. We found that our serum samples did contain bi-specific IgG4 antibodies (Figures 5B,C). We also found that more antibodies bound both DENV and *Ae. aegypti* SP than *Ae. albopictus* SP (Figures 5B,C). However, this was expected, as the individuals in the study had been exposed longer to *Ae. aegypti*. We are currently isolating the hybrid IgG4 molecules from these samples to test their effect on DENV infection.

Our experiments suggest the existence of IgG4 molecules binding DENV and SP, and they are an approximation of the effect of immunity against mosquito saliva in viral infections. More specific and accurate testing must be done to evaluate single bi-specific IgG4 antibodies and demonstrate the role of hybrid IgG4 molecules in vector-transmitted diseases.

## Concluding Remarks

It is of pivotal importance to determine whether there is production of saliva-pathogen IgG4 bi-specific antibodies during human infection with vector-borne pathogens, as well as the SPs that are involved in such a response. We speculate that in order to reach protective immunity against diseases, such as malaria through vaccination, treatment options should induce and resemble the natural immunity-building experience undergone by individuals living in areas with intense malaria transmission.

The study of the role of mosquito saliva immunity and antibodies against both pathogen and vector, as a tool to evaluate and monitor the exposure to infected mosquito bites, would improve control interventions and guide new strategies for protection and elimination. We have presented here evidence that IgG4-mediated responses are present in vector-borne diseases and that both arthropod saliva and the immune response against it may favor the formation of pathogen/mosquito bi-specific IgG4 antibodies. We hypothesize that bi-specific IgG4 is formed during chronic exposure to both mosquito bites and pathogen infection. Extensive additional research is necessary to characterize the nature of bi-specific antibodies and what role they may play in pathogenesis of or protection against vector-borne diseases.

## Ethics Statement

A written approval for collection of human samples was approved by Universidad de Pamplona, Los Patios Hospital and the Ethics Review Board of Hospital Erasmo Meoz.

## Author Notes

Dr. Londono-Renteria is a Research Associate at Dr. Colpitts’ Laboratory. This laboratory focuses on Dengue infection and pathogenesis studies as well as research on mosquito factors involved in the enhancement of transmission of vector-borne diseases.

## Author Contributions

BL-R, AT, and TC were involved in the bibliography search and the manuscript writing.

## Conflict of Interest Statement

The authors declare that the research was conducted in the absence of any commercial or financial relationships that could be construed as a potential conflict of interest.

## Abbreviations

ADE, antibody mediated enhancement; Ae, Aedes; BR1, B regulatory 1 cells; CL, cutaneous leishmaniasis; DENV, dengue virus; EBV, Epstein–Bar virus; FcR, fragment crystalizable region receptor; FS, Fogo Selvagem; gSG6-P1, salivary gland protein-6 peptide 1; HIV, human immunodeficiency virus; Ig, immunoglobulin G (IgG) or E (IgE).; L, Leishmania; Lu, Lutzomyia; ML, mucosal leishmaniasis; Ph, phlebotomus; PKDL, post Kala-Azar dermal leishmaniasis; SP, salivary protein; VL, visceral leishmaniasis.
